# Executive control processes are associated with individual fitness outcomes following regular exercise training: blood lactate profile curves and neuroimaging findings

**DOI:** 10.1038/s41598-018-23308-3

**Published:** 2018-03-20

**Authors:** M. C. Pensel, M. Daamen, L. Scheef, H. U. Knigge, S. Rojas Vega, J. A. Martin, H. H. Schild, H. K. Strüder, H. Boecker

**Affiliations:** 1 Functional Neuroimaging Group, Department of Radiology, University Hospital Bonn, Bonn, Germany; 2 Institute of Movement and Neurosciences, German Sport University Cologne, Cologne, Germany; 3 Department of Radiology, University Hospital Bonn, Bonn, Germany

## Abstract

Cardiovascular training has been associated with neuroimaging correlates of executive control functions (ECF) in seniors and children/adolescents, while complementary studies in middle-aged populations are lacking. Ascribing a prominent role to cardiorespiratory fitness improvements, most studies concentrated on training-induced gains in maximal oxygen uptake (VO_2_max), although other fitness indices may provide complementary information. Here, we investigated the impact of long-term sub-maximal exercise training on interference control, considering individual training-induced shifts in blood lactate profile curves (BLC) and VO_2_max. Twenty-three middle-aged sedentary males (M = 49 years) underwent a six-month exercise program (intervention group, IG). Additionally, 14 individuals without exercise training were recruited (control group, CG, M = 52 years). Interference control was assessed before and after the intervention, using a functional magnetic resonance imaging (fMRI) flanker paradigm. Task performance and brain activations showed no significant group-by-time interactions. However, regression analyses in the IG revealed significant associations between individual fitness gains and brain activation changes in frontal regions, which were not evident for VO_2_max, but for BLC. In conclusion, training-induced plasticity of ECF-related brain activity can be observed in late middle adulthood, but depends on individual fitness gains. For moderate training intensities, BLC shifts may provide sensitive markers for training-induced adaptations linked to ECF-related brain function.

## Introduction

Human studies indicate that exercise training improves executive control functions (ECF) necessary for ‘top-down’ regulation of goal-directed behaviour, and may help to ameliorate ageing-related decline of these cognitive abilities^1,2^ One aspect of ECF frequently examined in exercise-related studies is interference control, as measured by Stroop colour-word tasks^3^, or variants of the Eriksen flanker task^4^. In these paradigms, selective attention has to be focused on a target stimulus (or stimulus feature, e.g. colour), while inhibiting interference due to involuntary processing of distracting contextual information^2^.

Frequently, training-induced effects on ECF-related performance and brain function are linked to improvements in cardiorespiratory fitness (CRF), which are supposed to trigger physiological adaptations in the brain, e.g. through neurotrophic mechanisms^5^. To date, most evidence for the hypothesis that behavioural efficiency of interference control is positively related to CRF is based on cross-sectional studies examining populations with varying levels of habitual physical activity^2,6^. However, there are also more direct findings from randomized controlled trials (RCT)^1,7–10^ suggesting training-induced behavioural improvements. This holds also true for neuroimaging studies which have started to unravel the brain mechanisms by which physical training and cardiovascular fitness changes are associated with ECF. A seminal functional magnetic resonance imaging (fMRI) study^9^ examining older adults observed that six months of aerobic exercise did not only improve cardiovascular fitness and reduce behavioural conflict for incongruent stimuli in a flanker paradigm, but also showed enhanced brain activations in the right middle frontal gyrus (MFG) and left superior parietal lobule as well as decreased activations in the dorsal anterior cingulate cortex (ACC). Subsequent RCTs in children and older adults provided further support for exercise-induced changes of brain activation in lateral fronto-parietal and ACC areas^10–12^ Meanwhile, there is still a paucity of studies examining young and middle-aged adult populations, especially in the neuroimaging literature^13–15^, suggesting that more empirical research is needed to make inferences about the beneficial effects of regular physical activity in the latter age range^16^. Although the beneficial influence of regular physical exercise on ECF may be most pronounced during the dynamic phase of brain maturation and ageing, subtle improvements may already be observed during middle age. At least, epidemiological studies suggest that higher levels of physical activity (or physical fitness) during midlife may be linked with better cognitive performance (or less cognitive decline) in later life, although evidence remains mixed^17,18^. Various RCTs report direct associations between training-induced fitness gains and changes in brain structure^19–23^ or function^10,24^. Some of the above-mentioned interventional studies report no significant group differences in fitness changes between exercise and control arms, yet observe linear associations with individual amounts of change in physiological fitness parameters^20,23^, which may indicate that beneficial effects of fitness training could be obscured by varying treatment responses of the participants.

It is important to note that previous studies have mostly employed physical fitness parameters that do not necessarily best reflect the physiological changes typically induced by the intensity and duration of the stated exercise intervention. As an example, the aforementioned study by Colcombe *et al*.^9^ assessed fitness using maximal oxygen uptake (VO_2_max; i.e. reflecting exercise performance at 100% heart rate), although the exercise training program used required walking up to 45 min with an exercise intensity of only 60–70% heart rate (HR) reserve. While VO_2_max is an important determinant of endurance exercise performance^25^ and provides a good method to assess the physiological change at a maximal exercise level, it is not necessarily the optimal parameter to characterize effects of moderate exercise intensity. Instead, measurements of changes in blood lactate may provide a more sensitive indicator for adaptations in this exercise training range. Analyses of blood lactate profile curves (BLC) do not play a major role in the neuroimaging literature as yet, although they are well established in sport sciences for determining cardiovascular fitness^26^. The inherent advantage of BLC is that they contain information about changes related to sub-maximal exercise, whereas VO_2_max focuses primarily on higher exercise intensities. In running, the highest VO_2_max values are typically found in elite 5,000 m runners, because these athletes are required to run at 94–98% of maximum heart rate for ~13 minutes^27,28^, while marathon runners run at lower training intensities (i.e. 65–80% maximum heart rate) for 2 hours or longer, and other physiological factors become relatively more important here^25^. Notably, the determinants of sub-maximal exercise performance are a combination of VO_2_max, O_2_ cost of exercising at sub-maximal speeds (i.e. exercise economy), and the BLC^27^. Exercise economy is determined by many physiological and biomechanical factors that contribute to exercise performance, and is measured to quantify energy utilisation while exercising at an aerobic intensity^29,30^. In sum, VO_2_max is less sensitive for monitoring changes in fitness associated with moderate intense physical activity. Additionally, VO_2_ values, when expressed relative to body mass, may be artificially influenced by reduced weight rather than improved cardiovascular fitness^31,32^. For these reasons, we consider the assessment of BLC a more appropriate method to reflect adaptations due to regular moderate sub-maximal exercise training, which can eventually be linked to neuroimaging data.

Based on the aforementioned lines of reasoning, we conducted a longitudinal exercise intervention in a cohort of sedentary middle-aged males, to further investigate possible effects of regular aerobic exercise on interference control (as assessed in an fMRI Flanker task paradigm) in this age group, and selected a moderate, sub-maximal aerobic exercise training intensity that followed international recommendations^33^. We hypothesized behavioural and imaging changes to reflect training induced fitness gains in a dose-dependent fashion. Crucially, to assess physiological changes that specifically reflect adaptations due to this moderate cardiovascular training, we employed cycle ergometry not only to obtain a traditional parameter of maximum performance (VO_2_max), but also to examine training effects on BLC. In addition to comparing this training intervention group with a non-intervention control group, our study aimed to investigate how individual gains in BLC and VO_2_max correlate with changes in flanker task performance and brain activation in ECF-related fronto-parietal networks, while assuming that BLC would show more robust associations in our participants.

## Methods

### General study outline

**Figure 1:**
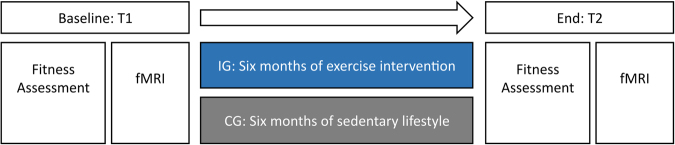
Experimental design. All participants were scanned before and after the intervention period and fitness assessments took place at T1 and T2.

The study design is summarized in Fig. 1. The experiment was part of a larger study that included a comprehensive battery of structural and functional magnetic resonance imaging sessions (to be reported elsewhere). A cohort of middle-aged sedentary males was recruited for a six-month exercise training intervention in preparation for a half marathon (intervention group, IG). We additionally recruited a control group (CG) not undergoing any systematic intervention. Group allocation was not randomized, i.e. the CG could primarily serve to monitor possible influences due to measurement repetition and scanner variance.

All participants completed a standardized fitness assessment (in Cologne) as well as structural and functional MRI protocols (in Bonn, average time lapse 30 ± 25.06 days), both at baseline (T1) and after the six-month period (T2). The participants of the intervention group started their regular training after the first neuroimaging scan and continued until the second scan was performed. To minimize acute influences of exercise training prior to assessments, all participants were instructed to avoid physical exercise for 24 hours before each MRI scan. All participants gave written informed consent. The study was approved by the local ethics committee of the University Hospital Bonn (Lfd. Nr. 297/08), according to national legislation and the Declaration of Helsinki, and is registered in the German Clinical Trials Register (Deutsches Register Klinischer Studien, DRKS, Study-ID: DRKS00013211, Universal Trial Number, UTN: U1111-1205-5292, date of registration: December 1^st^ 2017).

### Participants

Healthy, male, community-dwelling volunteers were recruited for the study. Exercise history was assessed based on semi-structured self-reports, and no subjects were included who reported at least weekly physical exercise training within the last two years. Furthermore, no participants were included who were involved in sport club activities during that time period. Subjects with daily physical activity related to personal transport (e.g. cycling to work) were excluded. Thirty-three participants joined the IG, of which 23 completed the study and were included in the final analysis (mean age 49.00 ± 5.32 years). Five participants dropped out from the intervention and for another five participants the fMRI data were corrupted. The initial control group included 25 participants of which 14 full datasets for imaging and behavioural parameters could be acquired (mean age 52.21 ± 6.39 years). From the control group, five participants dropped out, three participants did not keep their sedentary lifestyle and the fMRI data of another three participants were corrupted.

### Background characteristics

At study entry, medical history was assessed in a structured interview, and participants with neurological, psychiatric or cardiovascular diseases were excluded. Self-reported right-handedness of the study participants was verified using the Edinburgh Handedness Inventory^34^ to assure that no left-handers were included into the study. Verbal IQ was estimated with a vocabulary test^35^. Both at T1 and T2, depressive symptoms were assessed using the Beck Depression Inventory^36^.

### Training intervention program

Participants in the IG underwent a cardiovascular training program, supervised by a professional running school (Kölner Ausdauer- und Laufschule®, KALS, Cologne, DE) and tailored to individual capabilities, based on the physical examinations. Participants were given specific instructions regarding duration and intensity of the training (i.e. time and heart rate range). The training sessions lasted ~90 minutes and were highly structured (warm-up, teaching for coordination and running technique, runner-specific muscle training, endurance-run training and a cool down period). The program comprised three phases: (1) an adaptation phase which focused on developing a perception of the running tempo, (2) a build-up phase in which endurance performance was maximized by increasing the duration of exercise, and (3) a stabilization phase in which the sustained running tempo and training intensity was optimized. Participants were advised to train three days per week. At least one session per week was executed in small groups under supervision and close monitoring of adherence, while the remaining sessions were performed on an individual basis. Adherence for these remaining sessions was regularly inquired by training instructors, and no relevant training omissions were reported. During the six months of the intervention, three seminars were offered to participants to maintain motivation and to give further guidance on training and nutrition. At the end of the intervention period all participants finished a half marathon (i.e. 21,097.5 m). In contrast, the CG participants were asked to maintain their normal sedentary lifestyle without regular exercise throughout the six-month observation period.

### Anthropometry and cardiovascular fitness

Physical examinations were conducted before and after the intervention period in all IG and CG participants to establish their anthropometry and fitness status. Body weight and height were determined for calculation of body mass index (BMI), and resting heart rate was measured. Moreover, all participants underwent a graded exercise test on a cycle ergometer (Ergoline, Bitz, Germany). Testing started with an initial load of 25 W which was increased in 25 W steps every two minutes until volitional exhaustion. Electro-cardiogram activity and heart frequency were registered continuously throughout testing, and blood pressure was recorded every two minutes. Two measures of cardiovascular fitness were derived: Breath-by-breath analysis of oxygen uptake (VO_2_) was assessed using an ergospirometer (ZAN, Oberthulba, DE) and averaged at 10 s intervals, to identify VO_2_max. Additionally, prior to and during exercise, capillary whole blood samples (20 µl, non-fasting state) were taken from the left hyperaemic earlobe and analysed for lactate concentration at the end of each 25 W stage, to derive BLC as an indicator for fitness adaptations reflecting moderate training. The samples were immediately placed in a haemolysing solution, and analysed in our Cologne laboratory (BIOSEN C-line; EKF, London, United Kingdom).

### Flanker task MRI paradigm

Participants performed a modified version of the Eriksen Flanker Task during fMRI acquisition: The paradigm involved the visual presentation of four kinds of stimuli, each consisting of five arrows. Participants were instructed to indicate, using their right hand, as fast as possible whether the central arrow pointed right (middle finger button) or left (index finger button). The central stimulus pointed either in the same (congruent: →→→→→ or ←←←←←) or in the opposite direction (incongruent: →→←→→ or ←←→←←) as the four surrounding flanker stimuli. Each trial began with a pre-cue presentation of the flanking stimuli (→→ →→ or ←← ←←) alone for 100 ms to facilitate conflict induction^37^, followed by the proper flanker stimulus shown for 1000 ms, and a fixation cross baseline. To introduce additional temporal jitter, randomized inter-trial intervals were used (range 3000 to 8000 ms). Each of the four stimulus types was presented 50 times, resulting in a total of 200 events in pseudo-randomized order (total duration ~ 19 minutes). The task was visualized with the software Presentation® (Neurobehavioral Systems Inc., Albany, USA). Stimuli were projected onto a display in the scanner room, visible through a mirror system mounted on the MRI head coil. Responses were recorded with an MRI-compatible button box (LUMItouch^TM^, Photon Control Inc., Burnaby, CA). In each fMRI run 430 T2*-weighted EPI volumes were acquired on a 3 T Philips Achieva system with an 8-channel sensitivity encoding (SENSE) head coil (Philips Medical Systems, Best, NL): TR = 2595 ms, TE = 35 ms, flip angle = 90°, SENSE factor** = **2, ascending interleaved acquisition of 41 axial slices, slice thickness = 3.6 mm (no gap), FOV = 230 × 230 mm, reconstructed isotropic voxel size = 3.6 mm^3^. Slices were oriented parallel to an axial plane intersecting the genu and splenium of the corpus callosum using SmartExam, an automated planning procedure, to minimize between-session differences. Two T1-weighted 3D-MPRAGE datasets (TI = 1300 ms, TR = 7.7 ms, TE = 3.9 ms, flip angle = 15°) were acquired with a 1 mm^3^ isotropic voxel resolution, realigned, and averaged for anatomical reference and spatial normalization.

### Data analysis

#### Cardiovascular fitness

Fitness levels were assessed by deriving BLC and VO_2_max from the graded exercise tests. To provide a simple measure that expresses fitness-related shifts in BLC, we calculated the respective area under the curve (AUC). Lactate values of all participants displayed a mono-exponential characteristic between 50 and 125 W at T1 and T2 (y = c × e^a^, overall R^2^ = 0.98 ± 0.02). Missing intermediate values were interpolated to the mono-exponential curve (four participants with one missing value each). We determined the AUC for every participant by applying a trapezoidal approximation method, Equation (1):

1 $${{\rm{A}}{\rm{U}}{\rm{C}}}_{50-125}={\rm{\Delta }}x/2\,[f(50\,{\rm{W}})+2f(75\,{\rm{W}})+2f(100\,{\rm{W}})+f(125\,{\rm{W}})]$$

An improvement in cardiovascular fitness is typically expressed by a rightward shift of the BLC, indicating lower blood lactate concentration for a given work rate, which is reflected by a decrease in AUC_50-125_. To account for individual baseline differences, fitness change was expressed as relative percentage value at T2 as compared to T1, Equation (2):

2 $${\rm{ \% }}d{{\rm{A}}{\rm{U}}{\rm{C}}}_{50-125}=[({{\rm{A}}{\rm{U}}{\rm{C}}}_{50-125}{\rm{T}}2-AU{C}_{50-125}{\rm{T}}1)/{{\rm{A}}{\rm{U}}{\rm{C}}}_{50-125}{\rm{T}}1]\,\times \,100$$

Note that *negative* values in %dAUC reflect *improvements* in fitness. To facilitate comparisons with previous studies (for example Colcombe *et al*. 2004)^9^, we computed a complementary %dVO_2_max score to operationalize training-induced changes in maximum oxygen uptake, Equation (3)

3 $${\rm{ \% }}dV{O}_{2}\,max\,=\,[({{\rm{V}}{\rm{O}}}_{2}\,max\,{\rm{T}}2-{{\rm{V}}{\rm{O}}}_{2}\,max\,{\rm{T}}1)/{{\rm{V}}{\rm{O}}}_{2}{\rm{m}}{\rm{a}}{\rm{x}}{\rm{T}}1]\,\times \,100$$

Here, fitness *improvements* are reflected by *positive* values.

#### Task behaviour

In flanker paradigms, interference effects are typically inferred from prolonged RT or decreased accuracy (Accy) rates for central target stimuli with incongruent (as compared with congruent) flanker stimuli, with smaller increases of RT and smaller decreases of Accy rates, respectively, suggesting better interference resolution (and, hence, higher efficiency of ECF). Accordingly, we computed the Accy of indicating the correct direction of the central stimuli, and measured the reaction times (RT) of valid responses. The relative increase in average RT for incongruent as compared to congruent stimuli was calculated, using the following established^9^ Equation (4)

4 $${\rm{ \% }}DiffRT=[({\rm{R}}{\rm{T}}{\rm{i}}{\rm{n}}{\rm{c}}-{\rm{R}}{\rm{T}}{\rm{c}}{\rm{o}}{\rm{n}})/{\rm{R}}{\rm{T}}{\rm{c}}{\rm{o}}{\rm{n}}]\,\times \,100$$

In an analogous manner, we also calculated the relative difference in Accy for incongruent as compared to congruent events, Equation (5):

5 $${\rm{ \% }}DiffAccy=[({\rm{A}}{\rm{c}}{\rm{c}}{\rm{y}}\,{\rm{i}}{\rm{n}}{\rm{c}}{\textstyle \text{-}}{\rm{A}}{\rm{c}}{\rm{c}}{\rm{y}}\,{\rm{c}}{\rm{o}}{\rm{n}})/{\rm{A}}{\rm{c}}{\rm{c}}{\rm{y}}\,{\rm{c}}{\rm{o}}{\rm{n}}]\,\times \,100$$

#### Statistical analysis of background, fitness and behavioural variables

Statistical analyses for background characteristics, anthropometric and fitness parameters as well as task behavioural data were performed using IBM SPSS Statistics Version 24® (Armonk, NY: IBM Corp). Variables examined at baseline only were compared using Student’s t-test. Longitudinal data were analysed using a mixed ANOVA, with group (IG, CG) as between-subject factor and time (T1, T2) as within-subject factor. Post-hoc comparisons between conditions were conducted with Student’s t-tests. Moreover, to test correlations between the individual amount of fitness gains in the IG and corresponding changes in task behaviour, Pearson correlations between the respective change scores were examined.

#### Functional imaging analysis

Our fMRI data were analysed with SPM8 (Wellcome Department of Imaging Neuroscience, London, UK: http://www.fil.ion.ucl.ac.uk/spm/). Preprocessing of the functional data included slice-time correction, realignment and unwarping, segmentation^38^ of the coregistered anatomical scans to derive parameters for subsequent normalization, and smoothing with an 8 mm full width at half maximum Gaussian kernel. For the first-level analysis, a general linear model^39^ was set up in an event-related design, with separate regressors for incongruent (inc) and congruent (con) stimuli followed by correct responses, and an additional nuisance regressor for errors and missing trials. The congruent and incongruent events were modelled by the flanker stimulus onsets, while each event duration was specified using the respective RT. Time series were convolved with the canonical hemodynamic response function (HRF). As additional nuisance variables, we included time and dispersion derivatives of the HRF, six motion parameters (rigid body translations and rotations) and an additional time course of the average signal from the white matter to the design matrix. This latter parameter was obtained by applying the white matter segmentation of each participant (value of probability threshold at 0.99), and reading out the mean signal time course within the white matter volume from the realigned and normalized functional images, using the *MarsBar* toolbox^40,41^. At first level, we calculated the interference contrast (inc > con) for T1 and T2, and included the contrast images in second-level group analyses in a repeated-measures flexible factorial design, with time (T1, T2) as a within-subject factor and group (IG, CG) and subject as between-subject factors^42^. Here, the group × time interaction contrast indicates differential activation changes from T1 to T2 between the study groups and, hence, exercise training effects on task-related brain activity. To explore linear associations between the individual extent of fitness-related gains and brain activation changes in the IG, regression analyses were performed, with the differential congruency contrast [(Inc > con T1) vs. (Inc > con T2)] as the dependent variable, and %dAUC_50-125_ and %VO_2_max, respectively, as predictor variables. Clusters were considered significant at p = 0.05 FWEc (cluster-level corrected), with a voxel-wise threshold of p < 0.001 uncorrected. Coordinates are reported in MNI (Montreal Neurological Institute) space. Anatomical locations in functional activation maps were determined using AAL^43^, as provided by the WFU Pick Atlas^44^. The datasets generated during and/or analysed during the current study are available from the corresponding author on reasonable request.

## Results

### Background characteristics

Group comparisons for background characteristics are summarized in Table 1. There were no significant between-group differences for age (t = −1.65, p = 0.11) and baseline IQ (t = 1.51, p = 0.14). Moreover there was no main effect of group (F(1, 35) = 1.97, p = 0.17) or time (F(1, 35) = 3.44, p = 0.07), nor a significant group × time interaction (F(1, 35) = 0.48, p = 0.49) for the BDI depression scores. Background characteristics of participants that dropped out or had incomplete datasets were not statistically different from those who were finally analysed (p values > 0.1).

**Table 1:** Background characteristics: ANOVAs for repeated measures (interaction factors: group, time) and t-tests. Significance level is set to p < 0.05. For exploratory post hoc t-tests of ANOVA results, significance levels were Bonferroni corrected and changed to p < 0.0125. Applied measures for effect sizes: t-tests: Cohen’s d; ANOVA: Partial eta². Parameters: Age, estimated Intelligence quotient (IQ), Beck Depression Inventory (BDI).

	T1	mean ±Standard deviation (SD)	T2	mean ±SD	Inference		Df	p	Effect size
**Age**	IG	49.00 ±5.32			T1: IG v CG	t = −1.65	35	0.11	−0.56
	CG	52.21 ±6.39							
**IQ**	IG	113.04 ±10.06			T1: IG v CG	t = 1.51	35	0.14	0.51
	CG	107.43 ±12.39							
**BDI**	IG	2.09 ±2.84	IG	1.30 ±1.92	T1: IG v CG	t = 0.98	35	0.34	0.33
					T2: IG v CG	t = 1.83	35	0.08	0.62
	CG	3.14 ±3.72	CG	2.79 ±3.02	IG: T1 v T2	t = −2.05	22	0.05	−0.43
					CG: T1 v T2	t = −0.75	13	0.47	−0.20
					Time:	F = 3.44	1,35	0.07	0.09
					Group:	F = 1.97	1,35	0.17	0.05
					Interaction	F = 0.48	1,35	0.49	0.01

### Anthropometry and cardiovascular fitness

There was no significant main effect of group (F(1,35) = 0.06, p = 0.81) but a significant main effect of time (F(1,35) = 7.28, p = 0.011), and a significant group × time interaction F(1,35 = 27,07, p < 0.001) for BMI. Moreover, there was a significant main effect of group (F(1,35) = 6.34, p = 0.017) and time (F(1,35) = 20.77, p < 0.001), and a significant group × time interaction F(1,35) = 12.7, p = 0.001) for resting heart rate. Post-hoc t-tests indicate that these interactions were driven by a BMI and resting heart rate reduction for the IG only (see Table 2).

**Table 2:** Anthropometry and cardiovascular fitness: ANOVAs for repeated measures (interaction factors: group, time) and t-tests. Significance level is set to p < 0.05. For exploratory t-tests, significance levels were Bonferroni corrected and changed to p < 0.0125. Applied measures for effect sizes: t-tests: Cohen’s d; ANOVA: Partial eta². Parameters: AUC_50-125_ [(mmol/l)*W] (CG n = 11), VO_2_max [ml/min] (CG n = 11), BMI [kg/m²], heart rate at rest [min^−1^].

	T1	Mean ±SD	T2	mean ± SD	Inference		Df	p	Effect size
BLC AUC_50-125_	IG	183.06 ±68.41	IG	132.96 ±52.72	T1: IG v CG	t = −1.72	31.85	0.095	−0.63
					T2: IG v CG	t = 3.64	32	0.001	1.33
	CG	154.12 ±29.58	CG	181.22 ±24.65	IG: T1 v T2	t = −5.11	22	<0.001	−1.07
					CG: T1 v T2	t = 2.63	10	0.025	0.79
					group	F = 0.30	1, 32	0.589	0.01
					time	F = 2.09	1, 32	0.158	0.06
					interaction	F = 23.51	1, 32	<0.001	0.42
VO_2_max	IG	3058.61 ±587.43	IG	3510.97 ±582.70	T1: IG v CG	t = −3.88	31.94	<0.001	−1.42
					T2: IG v CG	t = −5.50	32	<0.001	−2.01
	CG	2477.35 ±286.37	CG	2458.91 ±354.57	IG: T1 v T2	t = 4.96	22	<0.001	1.03
					CG: T1 v T2	t = −0.30	10	0.771	−0.09
					group	F = 7.97	1, 32	0.008	0.20
					time	F = 3.84	1, 32	0.069	0.10
					interaction	F = 7.04	1, 32	0.012	0.18
BMI	IG	27.15 ±2.81	IG	26.16 ±2.71	T1: IG v CG	t = −0.91	35	0.369	−0.31
					T2: IG v CG	t = 0.43	35	0.668	0.15
	CG	26.26 ±3.05	CG	26.57 ±3.03	IG: T1 v T2	t = −5.90	22	<0.001	−1.23
					CG: T1 v T2	t = 1.92	13	0.077	0.51
					group	F = 0.06	1, 35	0.805	0.002
					time	F = 7.30	1, 35	0.011	0.78
					interaction	F = 27.07	1, 35	<0.001	0.44
heart rate at rest	IG	76.57 ±9.96	IG	64.26 ±8.00	T1: IG v CG	t = 0.48	35	0.636	0.16
					T2: IG v CG	t = 3.95	35	<0.001	1.34
	CG	78.07 ±8.04	CG	76.57 ±10.93	IG: T1 v T2	t = −6.93	22	<0.001	−1.45
					CG: T1 v T2	t = −0.58	13	0.569	−0.16
					group	F = 6.34	1, 35	0.017	0.14
					time	F = 20.77	1, 35	<0.001	0.34
					interaction	F = 12.72	1, 35	0.001	0.27
lactate at rest	IG	1.02 ±0.40	IG	0.94 ±0.33	T1: IG v CG	t = −1.35	32	0.185	−0.50
					T2: IG v CG	t = 0.47	32	0.640	0.17
	CG	0.84 ±0.20	CG	1.00 ±0.24	IG: T1 v T2	t = −0.91	22	0.371	−0.19
					CG: T1 v T2	t = 2.52	10	0.030	0.76
					group	F = 0.34	1, 32	0.562	0.01
					time	F = 0.43	1, 32	0.515	0.01
					interaction	F = 3.37	1, 32	0.076	0.10

**Figure 2:**
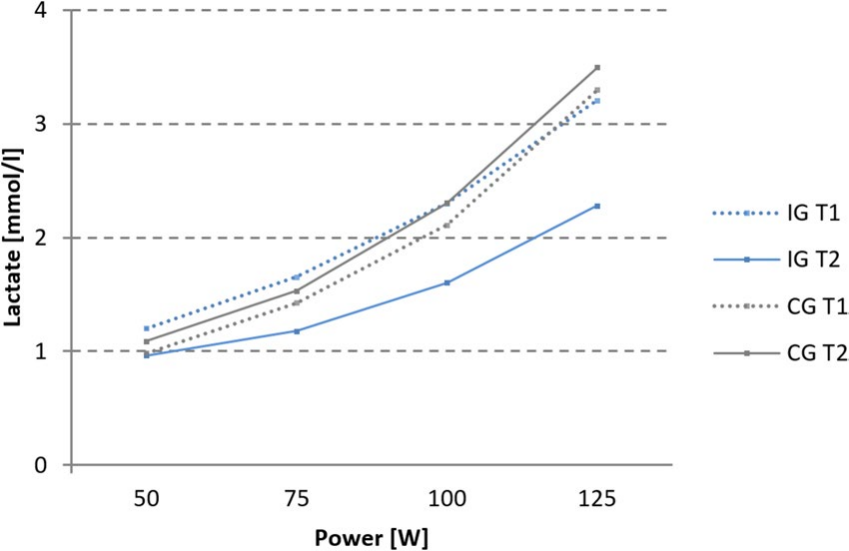
Blood lactate profile curves: BLC for IG (n = 23) and CG (n = 11) at T1 and T2 (50–125 W, group mean values).

**Figure 3:**
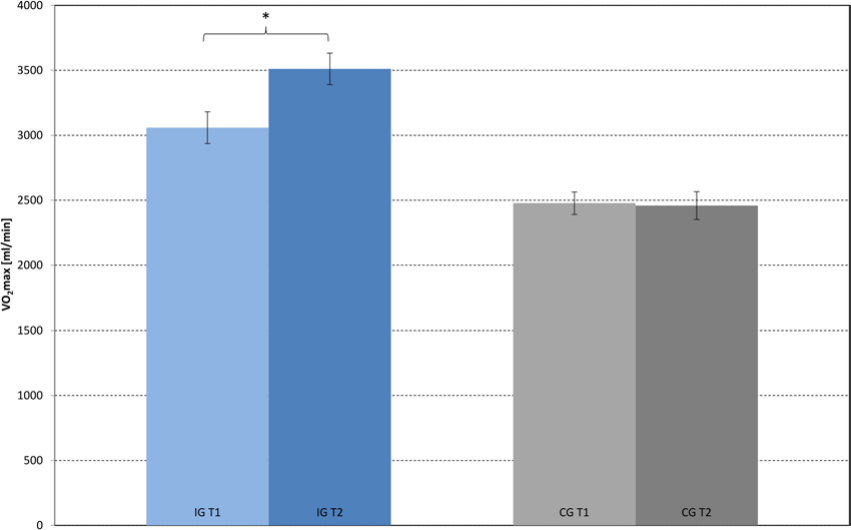
Maximal oxygen uptake: IG (n = 23) and CG (n = 11): VO_2_max-values at T1 and T2. Group mean values and standard errors.

Complete exercise physiological data were available for all 23 IG, and 11 CG participants. As a result, group comparisons had to be restricted to reduced CG samples for these variables. Lactate at rest was not significantly different between the two groups or time points. There was no significant main effect of group (F(1,32) = 0.30, p = 0.59) or time (F(1, 32) = 2.09, p = 0.158), yet a significant group × time interaction F(1, 32 = 23.51, p < 0.001) for BLC AUC_50-125_. There was a significant main effect of group (F(1,32) = 7.97, p = 0.008) but not of time (F(1,32) = 3.84, p = 0.069) for VO_2_max. The main effect of group was qualified by a significant group × time interaction F(1,32 = 7.04, p = 0.012). Post-hoc comparisons indicate that the IG showed a positive change in both fitness parameters with a significant decrease of AUC_50-125_ (Fig. 2) and increase of VO_2_max (Fig. 3), yet without a significant correlation between these changes (r = −0.076, p = 0.730). Meanwhile, there was no significant change for AUC_50-125_ (Fig. 2) or VO_2_max in the CG (Fig. 3).

### Behavioural performance

Considering the behavioural interference scores (i.e. the relative increase of RT and decrease of Accy for incongruent as compared to congruent stimuli, see Table 3), there was no significant main effect of group (F(1, 35) = 2.55, p = 0.120) or time (F(1, 35) = 1.78, p = 0.190), and no significant group × time interaction (F(1, 35) = 2.25, p = 0.142) for %DiffAccy. Moreover, there was no significant main effect of group (F(1, 35) = 1.66, p = 0.205) and again no significant group × time interaction (F(1, 35) = 0.21, p = 0.650) for %DiffRT, but a significant effect for time (F(1, 35) = 7.20, p = 0.011). In the IG, there were no significant correlations for relative changes of AUC_50-125_ with %DiffRT (Pearsons r = 0.061, p = 0.781) or with %DiffAccy (r = 0.244, p = 0.261). In a similar manner, for relative changes of VO_2_max, there were neither a significant correlation with %DiffRT (r = 0.075, p = 0.734) nor with %DiffAccy (r = 0.273, p = 0.207).

**Table 3:** Flanker task: Accuracy and reaction times. ANOVAs for repeated measures (interaction factors: group, time) and t-tests. Significance level is set to p < 0.05. For exploratory t-tests, significance levels were Bonferroni corrected and changed to p < 0.0125. Applied measures for effect sizes: t-tests: Cohen’s d; ANOVA: Partial eta². Parameters: %DiffAccy [%], %DiffRT [%].

	T1	**Mean** **±SD**	T2	**Mean** **±SD**	**Inference**		**Df**	**p**	**Effect size**
%DiffAccy [(inc-con)/con] × 100	IG	−1.57 ±2.47	IG	−1.48 ±1.99	T1: IG v CG T2: IG v CG	t = 2.12	35	0.042	0.72
						t = 0.15	35	0.880	0.05
	CG	0.10 ±2.08	CG	−1.37 ±2.45	IG: T1 v T2	t = 0.16	22	0.877	0.03
					CG: T1 v T2	t = −1.50	13	0.158	−0.40
					group	F = 2.55	1, 35	0.120	0.68
					time	F = 1.78	1, 35	0.190	0.05
					interaction	F = 2.25	1, 35	0.142	0.06
%DiffRT [(inc-con)/con] × 100	IG	17.34 ±6.49	IG	14.66 ±4.36	T1: IG v CG	t = 0.84	35	0.408	0.28
					T2: IG v CG	t = 1.68	35	0.101	0.57
	CG	19.28 ±7. 37	CG	17.38 ±5.40	IG: T1 v T2	t = −2.48	22	0.021	−0.52
					CG: T1 v T2	t = −1.49	13	0.161	−0.40
					group	F = 1.67	1, 35	0.205	0.05
					time	F = 7.20	1, 35	0.011	0.17
					interaction	F = 0.21	1, 35	0.650	0.01

### Imaging

**Figure 4:**
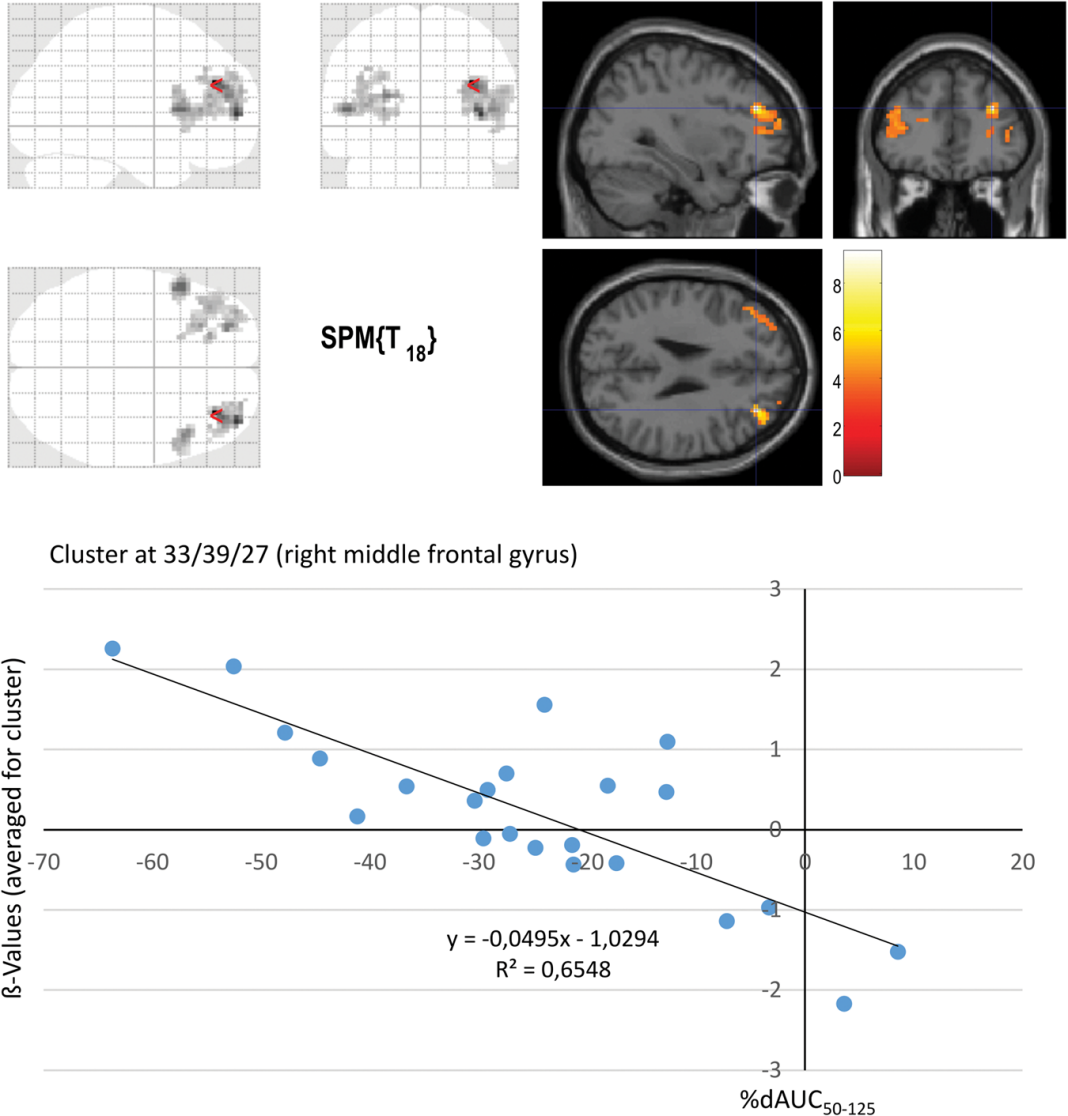
Imaging: regression %dAUC_50-125_: IG (n = 23): Negative correlation: [%dAUC_50-125_] × [(inc > con T1) < (inc > con T2)]. p = 0.001, clusters surviving FWEc-correction at 0.05, threshold = 57 voxels. Crosshairs in global maximum (right middle frontal gyrus).

An ANOVA for the interference contrast, i.e. (inc > con T1) × (inc > con T2), for IG (n = 23) vs CG (n = 14) showed no significant FWEc-corrected results for the main effects of group, time, or group × time interaction. However, using %dAUC_50-125_ as predictor variable (and age, estimated IQ and change in BDI as covariates) in a regression analysis within the IG, significant associations with brain activation changes were obtained in right middle frontal gyrus, left inferior frontal gyrus, left middle frontal gyrus, left Insula and right inferior frontal gyrus (see Table 4 and Fig. 4). These results indicate that those IG participants with stronger fitness increases, as expressed by more negative %dAUC_50-125_ values, showed stronger activation increases over time in the aforementioned areas. In the respective regression analysis using VO_2_max as a predictor variable, no significant clusters emerged.

**Table 4:** Imaging findings Regression %dAUC_50-125_. IG: Negative correlation: [%dAUC_50-125_] × [(inc > con T1) < (inc > con T2)]. p = 0.001. Results are shown for clusters comprising at least five voxels. Marked* clusters survive FWEc = 0.05, threshold = 57 voxels.

Brain regions	cluster Size	z-score (cluster peak)	MNI-coordinates cluster peak		
			X	Y	Z
Right middle frontal gyrus	185*	5.57	33	39	27
Right middle frontal gyrus		5.08	39	54	6
Right middle frontal gyrus		3.95	27	57	21
Left inferior frontal gyrus, pars opercularis	57*	4.65	−51	18	9
Left middle frontal gyrus	239*	4.42	−24	48	18
Left Insula		4.15	−27	30	9
		4.02	−21	36	18
Right Precuneus	34	4.35	9	−69	51
Right inferior frontal gyrus, pars opercularis	106*	4.34	48	18	9
Right inferior frontal gyrus, pars opercularis		3.88	54	12	21
		3.59	48	18	−6
Left superior temporal gyrus	10	4.11	−66	−36	9
Left middle temporal gyrus		3.31	−57	−33	3
Left Precuneus	21	4.03	−12	−63	54
Right middle occipital gyrus	21	3.99	30	−96	3
Right inferior occipital cortex		3.53	36	−87	−12
Left supramarginal gyrus	24	3.97	−54	−48	27
Left supramarginal gyrus		3.71	−57	−42	33
Right inferior temporal gyrus	15	3.95	57	−54	−6
Right superior occipital gyrus	41	3.92	24	−75	45
Right angular gyrus		3.71	33	−69	45
Right middle occipital gyrus		3.38	33	−75	36
	16	3.85	30	21	9
	16	3.76	−24	−51	−33
Left lobule VI of cerebellar hemisphere		3.65	−24	−60	−33
Right middle occipital gyrus	20	3.58	39	−84	18
Right middle occipital gyrus		3.52	45	−78	18
Right middle occipital gyrus		3.43	45	−78	3
Left crus I of cerebellar hemisphere	8	3.55	−39	−54	−39
Left middle occipital gyrus	5	3.55	−27	−90	−3
Left inferior frontal gyrus, pars triangularis	10	3.47	−51	39	−3
Left inferior frontal gyrus, pars triangularis		3.28	−45	33	−3

## Discussion

We investigated the impact of a six-month exercise training intervention on flanker task performance in a middle-aged cohort of sedentary males. To assess fitness improvements, we investigated not only VO_2_max as a traditional parameter of maximum performance but also measured changes in BLC, which we expected to account better for the specific physiological changes related to our moderate intensity training. While the IG as a whole showed no significant behavioural and neuroimaging differences compared to a passive CG, regression analyses within the IG suggested that brain activation changes were positively correlated with the extent of individual training-related fitness gains. Critically, significant associations were observed for BLC, but not for VO_2_max, and were concentrated in bilateral frontal regions, consistent with (and extending) findings of exercise-related fMRI studies using ECF paradigms in children and older adults. Our data suggest that our BLC-based measure is more sensitive than maximum oxygen uptake in detecting brain activation changes related to fitness gains after moderate exercise training. In the following, we will discuss our results point by point in further detail.

Based on previous fMRI publications examining exercise interventions on ECF^9,45^, we expected training-related changes in neuronal activity for incongruent relative to congruent flanker stimuli in fronto-cingulo-parietal networks, which are frequently implicated in executive functions in general^46^. The IG as a whole showed no significant activation changes in comparison to a passive CG, although we cannot exclude that subtle group by time interactions existed, which remained undetected due to a limited final sample size and hence, statistical power. However, we found evidence for our assumption that the amount of individual training-induced fitness gains (as measured by AUC_50-125_) in the IG was related to activation changes in some aspects of these brain networks in a linear manner. Previous fMRI studies using ECF-related tasks have already reported cross-sectional linear associations between fitness status and brain activation levels^47,48^, but to the best of our knowledge, our findings are novel in that they link training-induced fitness improvements with ECF-related brain activation changes. Although correlative, this observation strengthens the idea that physical training interventions may influence ECF-related brain functions via improvements in CRF, and there are signs that shifts in BLC are better reflecting these improvements, as do changes in VO_2_max^49^. Nonetheless, our blood lactate-based measure suffers from similar inferential limitations. Both parameters are primarily determined by peripheral (especially: muscle) physiology, and there is no compelling reason to assume that there is a one-by-one correspondence of adaptations on the brain level (as already pointed out by Dustman^50^ regarding VO_2_max). Our measure only provides a proxy for underlying physiological adaptations that are supposed to trigger neuro-humeral mechanisms on the brain level, which ultimately induce functional plasticity. These mediating processes still need to be elucidated, although there are preliminary clues for a modulatory influence of lactate in the brain: Most notably, it was suggested that lactate-related signalling mechanisms could influence the secretion of brain derived neurotrophic factor (BDNF)^51,52^, which is likely involved in the positive effects of physical exercise on brain and cognitive function^5^. Therefore, it can be speculated that training-induced adaptations of brain lactate signalling may also modulate this plasticity-related mechanism, and have a distal effect on brain activity during cognitive (e.g. ECF) tasks, although further research is necessary to corroborate this idea.

The relevant brain regions show partial overlap with previous cross-sectional and interventional exercise studies using flanker tasks or similar ECF paradigms. We observed linear associations with AUC_50-125_ in bilateral MFG and IFG regions, which is in line with findings in several exercise-related investigations showing training-related activation changes in bilateral^45,47^ or right frontal areas^9,48^. Furthermore, we found linear associations with training-related activation changes in the left anterior insula, an area which has also been connected to ECF processing^53–55^. Some studies have additionally reported fitness-related differences in ACC activation^9,10,12,45,48^. In contrast, we found no significant group differences or linear associations with individual fitness gains in this region, not even at a liberal statistical threshold of p < 0.001 uncorrected.

It should be noted that abovementioned results are not in line with all parts of the existing literature. While our findings suggest *increased* recruitment of frontal regions with improved fitness^9^, some studies reported *lower* frontal activation in ‘fitter’ participants, both in cross-sectional^56,57^ and interventional^10,11^ studies, suggesting higher neural efficiency during task processing. For the ACC, both fitness-related increases^12,45,48^ and decreases^9,10^ of task-related brain activations have been reported, while in our study no change was observed. These mixed findings cannot easily be explained by obvious differences in study design (i.e. observational versus interventional studies) or age range (i.e. varying effects of physical exercise in different developmental stages). However, we also cannot discard that differences in task design might indeed play a role, e.g. by implicitly triggering different task processing strategies. Actually, there is some evidence that the physical fitness level is differentially related to performance in paradigms emphasizing proactive vs reactive control strategies^58,59^. As yet, the specific interrelations between exercise and neural correlates of ECFs are not entirely understood and, thus, require further investigation.

We did not find any significant exercise-related group effects in task performance, neither regarding RT nor Accy. While cross-sectional studies reported differences between high fit and low fit participants^9^, exercise-related interaction effects for reaction time differences between congruent and incongruent trials over time were not described^9,45^. For Accy, a previous study showed that a group of higher fit participants were able to maintain their Accy rates over time, while a group of lower fit participants did not^45^. The absence of significant behavioural interaction effects in our study may have several reasons. One explanation might be that existing behavioural effects are small, and could not be reliably detected due to the comparably small and uneven sample size^60^. In general, a recent meta-analysis^61^ suggested that the effect sizes of cardiovascular exercise on ECF are subtler than initially assumed^1^. In principle, effect sizes for pre-post changes of RT (Table 3) suggest at least a medium reduction of interference scores in the IG (d > 0.5), however, a small effect size was also observed in the CG (d > 0.2). As also indicated by the significant main effect of time in the corresponding ANOVA, we cannot discard the influence of mere repetition effects. Another possibility could be that the intensity (moderate) and duration (six months) of our exercise training was not sufficient to elicit clear behavioural effects, at least in this late middle-aged population where age-related decay of brain functions is only beginning.

As expected, our moderate-intensity physical exercise intervention was successful in increasing cardiovascular fitness of the intervention group, while no significant changes were observed in the passive control group, for both VO_2_max and AUC_50-125_, although it needs to be acknowledged that only a reduced CG sample was available for these group analyses. On the other hand, we have only detected significant correlations of brain activations during flanker task performance with changes in AUC_50-125_, but not in VO_2_max. Another study also failed to find such a correlation with VO_2_peak^12^. This may indicate that BLC shifts can better capture training-related changes, at least for moderate exercise regimen. The AUC_50-125_ value is easy to compute from the mono-exponential increase of the BLC. The first part of the curve (0–50 W) was neglected as we had no specific interest in the initial plateau or even negative dip of lactate concentration, typically observed at exercise onset from rest, but rather focused upon the subsequent increase. A key advantage of BLC is that it is less dependent on the ability (or motivation) of participants to reach their physiologically possible performance maximum, as the information is derived from shifts over a wider range of exercise intensities. This is crucial for the assessment of untrained participants who are not familiar with exercising at maximum capacity. Additional investigations are needed to further compare different fitness parameters and their utility in neuroimaging studies.

While we compared our IG with a passive control group, other studies have emphasized the importance of incorporating an active control condition such as ‘stretching and toning’^9^ or ‘stretching and coordination’ training^62^. It must be acknowledged that these training regimes may also influence executive control processing^10^, and may therefore mask existing effects of aerobic exercise when used as a control condition. Another important point to be considered in this context is that active control conditions are indeed suitable for controlling social interaction effects, however, allocation to a less demanding control condition like ‘stretching and toning’ may influence participants’ behaviour to independently modify lifestyle habits towards increased physical activity^22,63^, potentially biasing study outcomes. The latter problem would also apply to a waiting control group design^11,64^, i.e. asking control participants to delay their intention to train for another six months.

Moreover, group allocation was not randomized, and we acknowledge this as a major limitation of this study. Indeed, it is generally advisable to employ randomized study designs, e.g. by randomly attributing volunteers to either exercise or stretching and toning control groups^1,9^. Such randomized allocation allows controlling for potential self-selection bias and for social interactions among participants, as well as between participants and instructors.

Nevertheless, it should be stressed that the abovementioned shortcomings do not invalidate our main findings, as they were actually not based on between-group differences, but on direct associations between individual training-induced fitness gains and brain activation changes within the IG. While this does not preclude that the observed associations were actually mediated by confounding changes in uncontrolled background variables, the present findings justify further research into dose-dependent influences of fitness training on executive functions and their neural substrates.

Our study cannot explain the inter-individual differences in training outcomes for the IG participants. There was no complete monitoring of individual training adherence and training intensity, which would be the most evident reason for the variable outcomes. Actually, some intervention studies report dose-response relationships between attendance rates and improvements in behavioural and neurophysiological indices of executive function^64^. Future investigations are recommended to monitor training participation more closely, for example by using modern tracking devices^65^. Furthermore, it is recommended to control for background activity^63^ and to investigate other important factors such as genetics^14,66,67^ and nutrition^68^. Finally, as this study only included male participants, the influence of possible sex specific differences needs to be investigated. The inclusion of male-only participants was due to overarching study design priorities that were not related to the Flanker task itself. We cannot exclude that training effects for female participants would have been different. Actually, there is evidence that female participants show larger cognitive improvements than males^1,69^.

In conclusion, our data support the idea that training-induced plasticity of ECF-related brain activity can also be observed in late middle adulthood, while suggesting that this functional plasticity will depend on training-induced individual fitness gains. For moderate intensity training programs, which do not primarily aim to improve maximal oxygen uptake, BLC may provide a more sensitive marker for the underlying fitness changes than VO_2_max.
